# Analysis of small-angle X-ray scattering data in the presence of significant instrumental smearing

**DOI:** 10.1107/S1600576715023444

**Published:** 2016-02-01

**Authors:** Johan Bergenholtz, Jeanette Ulama, Malin Zackrisson Oskolkova

**Affiliations:** a Department of Chemistry and Molecular Biology, University of Gothenburg, SE-412 96 Göteborg, Sweden; b Division of Physical Chemistry, Center of Chemistry and Chemical Engineering, Lund University, SE-221 00 Lund, Sweden

**Keywords:** small-angle X-ray scattering, pinhole collimation, instrumental smearing effects

## Abstract

A laboratory-scale small-angle X-ray scattering instrument with pinhole collimation has been used to assess smearing effects due to instrumental resolution. A new, numerically efficient method to smear ideal model intensities is developed and presented. It allows for directly using measured profiles of isotropic but otherwise arbitrary beams in smearing calculations. Samples of low-polydispersity polymer spheres have been used to show that scattering data can in this way be quantitatively modeled even when there is substantial distortion due to instrumental resolution.

## Abstract 2

A new, numerically efficient method has been developed to account for instrumental resolution effects on isotropic small-angle X-ray scattering with pinhole collimation.

## 1. Introduction   

Quantitative modeling of small-angle scattering data is a powerful methodology for obtaining information about structure and interactions for a wide variety of colloidal dispersions and macromolecular solutions. Instrumental smearing effects, originating from limitations in instrumental resolution, can in many instances complicate matters. If not properly taken into account, such effects can lead to errors in the parameters extracted from the analysis (Hansen & Pedersen, 1991 ▸; Rennie *et al.*, 2013 ▸), or, if data are severely smeared, they can prevent quantitative analysis altogether. Smearing effects are well known in connection with instruments for small-angle X-ray scattering (SAXS) relying on long slit geometries (Glatter & Kratky, 1982 ▸; Feigin & Svergun, 1987 ▸; Fritz & Bergmann, 2006 ▸) and in small-angle neutron scattering (Barker & Pedersen, 1995 ▸). In the former the smearing is caused by collimation effects and in the latter the wavelength spread of the incident neutron beam usually strongly contributes in addition. With SAXS at synchrotrons, the high photon flux allows for using finely collimated beams. Such pinhole collimation is also becoming increasingly more common for laboratory-scale SAXS instruments. Instrumental smearing effects are generally considerably smaller for pinhole collimation and they are often neglected in the analysis of SAXS data. While this might be justified for many measurements at synchrotrons, it may or may not be so for laboratory-scale SAXS setups.

Even though recent developments in X-ray source technology and optics have resulted in remarkable increases in the beam flux of these laboratory-scale SAXS instruments (Skarzynski, 2013 ▸), resolution is still often sacrificed in favor of an increased flux to reduce measurement times. In addition, collimation lengths are kept fairly short for compactness, placing some restrictions on the maximum resolvable length scale. As a case in point, Wagner *et al.* (2000 ▸) collected scattering data using a home laboratory SAXS instrument on relatively monodisperse spherical particles as large as about 140 nm. They could only analyze their data quantitatively once collimation effects were accounted for by smearing the theoretical intensity using a calculated beam profile.

In the following we revisit the instrumental smearing problem due to finite collimation because with newly developed detectors direct imaging of the primary X-ray beam is possible. Thus, direct detailed characterization of the smearing due to collimation effects is also possible. Given that such smearing effects are usually accounted for using theoretical calculations of beam profiles (Miller *et al.*, 1984 ▸; Mildner & Carpenter, 1984 ▸; Ramakrishnan, 1985 ▸; Schmidt, 1988 ▸; Pedersen *et al.*, 1990 ▸) or Monte Carlo simulations (Lai & Cerrina, 1986 ▸; Pedersen *et al.*, 1990 ▸; Pedersen, 1993 ▸, 2004 ▸; Harris *et al.*, 1995 ▸), it becomes of interest to investigate how these methods compare when confronted with experimentally measured beam profiles. In addition, as already remarked upon, consideration of such effects is warranted, especially for laboratory-scale SAXS setups, and might expand the length scale regime that can accommodate a quantitative structural analysis.

We begin by briefly reviewing the finite-collimation smearing problem, the resolution function for radially symmetric data and the use of Gaussian functions. We then present an alternative way of dealing with the smearing effect, which incorporates directly the measured beam profile. This approach can be applied for radially symmetric but otherwise arbitrary beam profiles, in a simple and numerically efficient scheme. The new method is tested using calculations based on simple trapezoidal beam profiles as well as direct fitting of measured beam profiles on SAXS data for latex systems in a number of different instrumental configurations with varying degrees of data smearing.

## 2. Experimental   

### 2.1. Latex systems   

Two colloidal latex systems, labeled L5 and L5I, of differently sized particles were employed in this study. The latices contain spherical particles with a core of poly(heptafluoro­butyl methacrylate) with a grafted steric layer of poly(ethyl­ene glycol) dispersed in 10 m*M* (3 m*M* NaN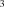 plus 7 m*M* NaCl) aqueous solution. Details of the emulsion polymerization procedure of the L5 system have been reported elsewhere and the L5 label has been kept the same as in the original work (Ulama *et al.*, 2014 ▸). The L5I system was synthesized according to the same protocol with the exception that it was carried out in a batch rather than semibatch synthesis. Specifically, all of the potassium persulfate initiator was added directly at the beginning of the synthesis instead of being fed slowly into the reaction mixture over a period of several hours. Whereas the semibatch synthesis of L5 produced a unimodal, quite monodisperse size distribution of particles, the batch procedure resulted in two size distributions. However, owing to settling of the largest particles (∼1 µm) only the smallest size distribution (radius ∼50 nm) is probed in the experiments.

### 2.2. SAXS measurements   

SAXS spectra were recorded at the Division of Physical Chemistry, Lund University, Sweden. The instrument is an automated SAXS pinhole system (Ganesha, JJ X-Ray A/S, Denmark) equipped with a high-brilliance microfocus sealed tube with shaped multilayer optics and a two-dimensional single-photon counting solid-state Pilatus detector (Dectris Ltd, Switzerland). Data were recorded using two- or three-pinhole collimation configurations and varying sample-to-detector distances as listed in Table 1 ▸. All configurations employed a scatterless aperture as the final aperture for the beam before it reaches the sample. Experimental data were processed and radially averaged using the *SAXSGUI* software, and the scattering spectra were obtained as a function of the magnitude of the scattering vector 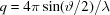, where ϑ is the scattering angle and λ is the wavelength (0.154 nm, Cu *K*α line). The scattering from the aqueous solvent in the same capillaries was measured as background and was subtracted to yield the excess scattering as a function of *q* for the latex samples. The scattering spectra from the samples were converted to an absolute scale using water as a standard. The primary beam was also directly imaged on the detector in the various configurations with an exposure time of 0.5 s. The two-dimensional scattering patterns of the beam were radially averaged in the same way as the sample spectra.

## 3. Collimation smearing   

### 3.1. Resolution function and the Hankel transform method   

Neglecting any wavelength spread of the beam, the ideal model intensity, 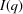, assumed to derive from an isotropically scattering sample, yields the smeared intensity, 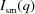, through a convolution with the beam intensity within the aperture image on the detector as (Miller *et al.*, 1984 ▸) 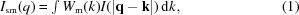
where the beam profile 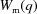 is normalized such that 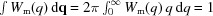. Equation (1) is equivalent to a description in terms of a resolution function, 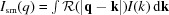 (Pedersen *et al.*, 1990 ▸), in this case assumed to be radially symmetric and dependent only on two-dimensional scattering vectors restricted to the plane of the detector. Fourier transformation of equation (1) leads to 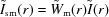, where the two-dimensional Fourier transform is given essentially by a zeroth-order Hankel transform (Ramakrishnan, 1985 ▸),
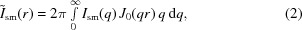
which involves the zeroth-order Bessel function of the first kind, 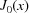. As the inverse transform is 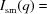 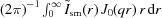, it follows that the smeared intensity can be determined from (Ramakrishnan, 1985 ▸; Bartlett & Ottewill, 1992 ▸) 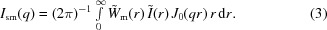
By changing the order of integration, the above result can be expressed as 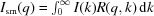, which is an often quoted result for radially averaged, isotropic scattering (Hjelm, 1988 ▸; Pedersen *et al.*, 1990 ▸; Pedersen, 1997 ▸; Pauw, 2013 ▸). Here, the resolution function is defined as 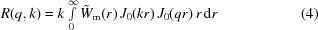
and, as outlined in Appendix *A* [App appa], it contains also the broadening due to the detector resolution.

Within this Hankel-transform formalism it is straightforward to derive a number of known results. For a perfectly collimated system the beam profile is given by a delta function, as 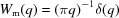, and the Hankel-transformed beam profile is unity. It follows that for the resolution function one obtains 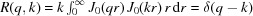 so that 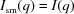. For a Gaussian beam profile, 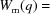 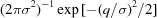, the Hankel transform is also a Gaussian function, 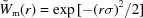. The corresponding resolution function follows directly from equation (4) as (Freltoft *et al.*, 1986 ▸) 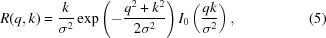
where 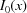 is the zeroth-order modified Bessel function of the first kind (Abramowitz & Stegun, 1972 ▸). With this result the smeared intensity is given by a single weighted integral over the ideal intensity as 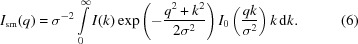
For 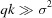, 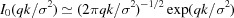, which when inserted into equation (6) leads almost to a Gaussian resolution function, 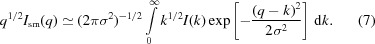
However, it is only when the factor 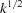 is replaced by 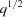 inside the integral that the resolution function becomes an actual Gaussian function for a Gaussian beam profile (Pedersen *et al.*, 1990 ▸).

As seen from the above, unless we employ a Gaussian representation of the measured beam profile, in which case we can start from equation (6) or perhaps one of the approximations to it, we are left with little alternative but to use equation (3) in accounting for smearing effects. This requires computing two Hankel transforms before completing the integral in equation (3). As an alternative, we formulate in the next section a new algorithm that accommodates arbitrary beam profiles in a much easier way.

### 3.2. Direct integration in polar coordinates   

**Figure 1:**
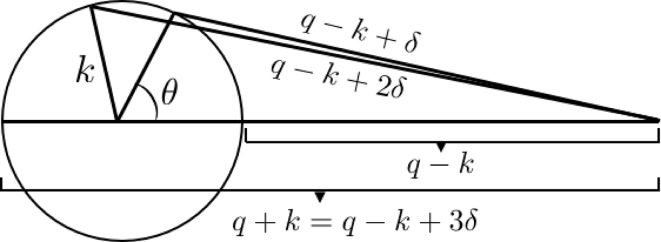
Illustration of the direct integration method employed. The circle represents the collimation image on the detector within which the radiation detected at *q* originates (Miller *et al.*, 1984 ▸). The ideal intensity is evaluated as a cubic (spline) polynomial with coefficients that vary depending on the interval. In this example, *s* has been divided into three intervals, corresponding to different angles θ. The first term in the integration in equation (11) starts from 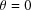, corresponding to 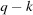, and extends to an angle that corresponds to 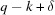. The next term is an integral between angles corresponding to 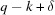 and 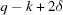. The final term is an integral between angles corresponding to 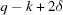 and 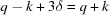, the latter of which makes an angle π.

In what follows, an efficient numerical algorithm is presented that solves equation (1) directly in polar coordinates for isotropic but otherwise arbitrary beam profiles. In polar coordinates equation (1) reads as 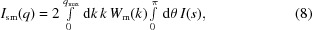
where 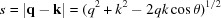 in terms of the angle θ between the vectors 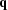 and 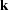. Furthermore, 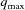 is a scattering vector beyond which the beam intensity is negligible. The troublesome square root in the argument of the ideal intensity in equation (8) can be avoided by regarding 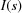 as a function of 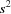 instead of *s* and letting it be represented as a piece-wise cubic spline:
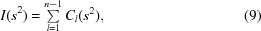
where 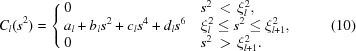
In the above, 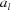, 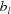, 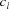 and 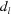 are the coefficients of the cubic spline representation of the intensity, which has been discretized using 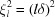. Next, referring to Fig. 1 ▸, we note that the inner integration over 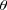 in equation (8) corresponds to the ideal intensity being sampled over the interval 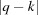 to 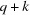. With *q* and *k* in equation (8) discretized using the same increment, 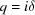 and 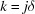, the inner integral is written as a sum of integrals, each one of which extends over θ values corresponding to one interval of the cubic spline: 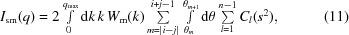
where the angles entering the integration limits are determined as 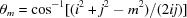. Again, they correspond by construction precisely to the nodes of the cubic spline. For instance, for 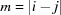, 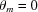 and for 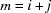, 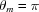. The inner integral can now be solved analytically, resulting in 
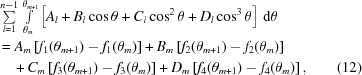
where 
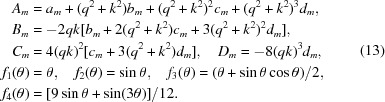
This direct integration algorithm is applied with just a single cubic spline fit of the model intensity, which converts the complicated *q* dependence of the model intensity into a simple one that allows for analytical completion of the inner of the two integrals in equation (8). A standard numerical routine for cubic splines (Press *et al.*, 1992 ▸), if necessary, can easily be modified to return the required arrays of coefficients, which allows for evaluation of equation (12). The remaining *k* integration in equation (11) is done using, for example, Simpson’s rule.

## 4. Results and discussion   

**Figure 2:**
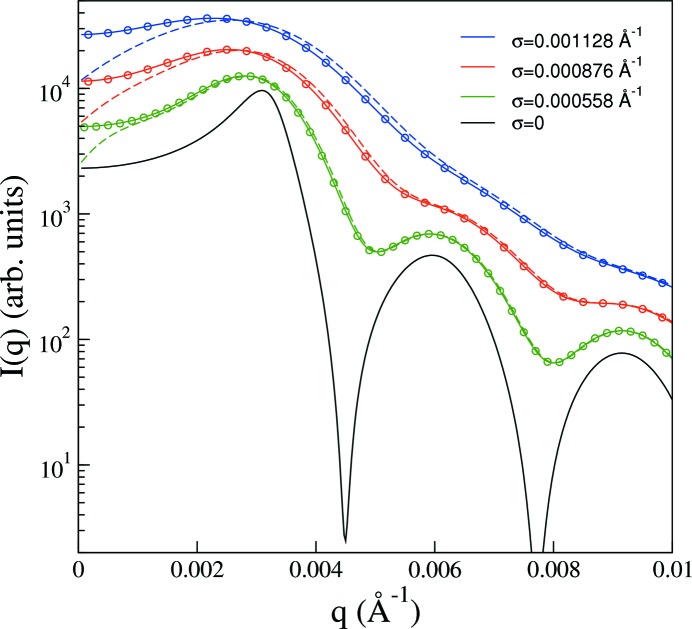
Model intensity (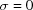) and resulting smeared intensities as a function of scattering vector for hard spheres of 100 nm mean radius at a volume fraction of 0.4 with 1% polydispersity included. The ideal intensity has been smeared using the two algorithms discussed in the text, one based on numerical integration of equation (6) (solid lines) and the other of equation (11) (symbols), both for Gaussian beam profiles characterized by standard deviation σ, as labeled. Results are also shown (dashed lines) for a Gaussian resolution function, *i.e.* 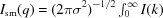
 × 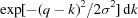. The results have been shifted along the intensity axis for clarity.

The direct integration algorithm is tested in Fig. 2 ▸ for Gaussian beam profiles of varying width using a model scattering function for slightly polydisperse (standard deviation divided by mean = 0.01) hard spheres (Griffith *et al.*, 1987 ▸) of 100 nm mean radius at a volume fraction of 0.4. As seen in Fig. 2 ▸, the structure factor peak is strongly smeared, as are the form factor minima. Clearly, features with a characteristic wavevector width approaching the width of the beam cannot be faithfully resolved. On the other hand, it is conceivable that such features can be captured indirectly through modeling if instrumental resolution effects are taken into account in an accurate way. This is the approach investigated here, rather than attempting to desmear experimental data (Glatter, 1977 ▸; Singh *et al.*, 1993 ▸; Bergmann *et al.*, 2000 ▸; Vad & Sager, 2011 ▸).

For Gaussian beam profiles the smearing algorithm based on equation (11) should yield the same result as a numerical integration of equation (6) for which a Gaussian beam profile was assumed at the outset. Fig. 2 ▸ demonstrates that the direct integration method indeed reproduces the result obtained from equation (6). In the figure results are also shown for a Gaussian resolution function, *i.e.* when 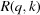 is assumed to be given by a Gaussian with a standard deviation equal to that of the Gaussian beam profile. In this case the smeared intensity is obtained as 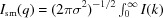
 × 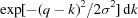, which is also shown in Fig. 2 ▸. This clearly produces different results from those corresponding to a Gaussian beam profile as obtained from equation (6). When the beam size is relatively small, corresponding to the smallest values of σ in Fig. 2 ▸, the difference between using the Gaussian beam profile and the Gaussian resolution function is comparatively small and occurs mostly for 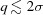, which is a region that is usually masked. However, in the presence of increased levels of smearing, *i.e.* for larger values of σ, the differences become more pronounced and extend over a relatively broad range of *q*, up to the first hump of the smeared form factor. As stressed by Pedersen *et al.* (1990 ▸), in modeling smearing effects, even though it is convenient to assume a Gaussian form for the radially averaged resolution function, particularly for small-angle neutron scattering (Bagger-Jörgensen *et al.*, 1997 ▸; Won *et al.*, 2000 ▸; Zackrisson *et al.*, 2005 ▸), it is important to employ equation (6). After all, it is just a single numerical integral that needs to be performed, and 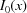 can easily be determined with high precision (Abramowitz & Stegun, 1972 ▸). Also, as shown here, an equally good alternative to using equation (6) is given by the direct integration method described in this work.

To investigate instrumental smearing effects experimentally it is important to use not only a well characterized model system but also one that produces sharp features in the scattering intensity under near-ideal conditions (Wignall, 1991 ▸). In this work, we use a thoroughly characterized latex system of fluorinated colloidal spheres, stabilized by grafted PEG polymer (Ulama *et al.*, 2014 ▸). The fluorination of the particles has a number of advantages, but in the present context the most important one is that it increases the electron density compared to fully hydrogenated polymers. The PEG graft contributes negligibly to the scattered intensity such that the particles can be modeled as homogeneous spheres.

**Figure 3:**
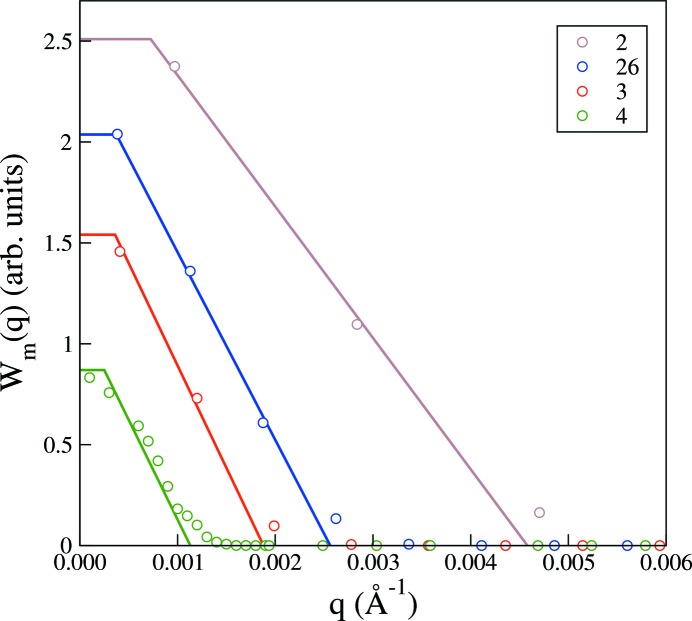
Experimentally measured beam intensity profiles, 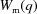, in arbitrary units, *versus* magnitude of the scattering vector, corresponding to the instrumental configurations of Table 1 ▸. The solid lines are calculated trapezoidal beam profiles.

The scattering from dilute dispersions of these particles was measured using an in house SAXS instrument in various two- and three-pinhole configurations as detailed in Table 1 ▸. From the geometry of the collimation system one can estimate the beam profile, which is, in lieu of a measurement of the beam profile, a common first step towards incorporating instrumental resolution effects. For instance, for uniformly illuminated apertures, one expects approximately a trapezoidal beam profile corresponding to the umbra and penumbra of the source image on the detector (Miller *et al.*, 1984 ▸). Although we expect a more complex beam profile, we find by comparison with the measurements of actual beam profiles shown in Fig. 3 ▸ that reasonable agreement is obtained if the diameter of the umbra on the detector, 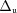, is determined by the first two pinholes and the diameter of the penumbra on the detector, 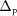, is set by the first and last of the pinholes, as 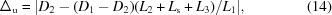


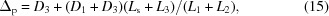
in terms of the diameters of the pinholes, 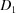, 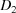 and 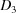, and the distances between them, 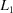 and 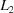, the distance from the final pinhole to the sample, 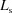, and the sample-to-detector distance, 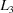. From the scattering angle corresponding to the full width of the beam, 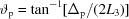, the resolution in wavevector space can be estimated as 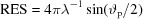, which is listed in Table 1 ▸. An improvement on the approximate trapezoidal beam profile is given by determining the area of overlap between the circular projections of the apertures on the detector (Barker & Pedersen, 1995 ▸).

**Figure 4:**
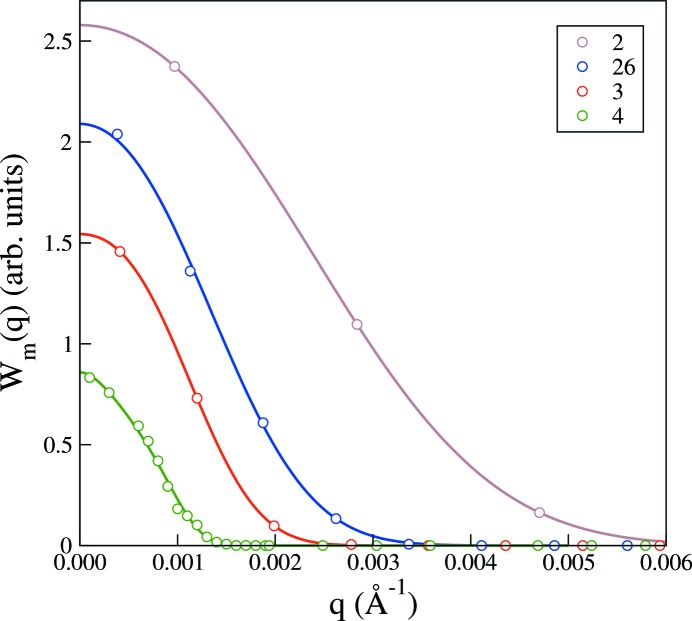
Experimentally measured beam intensity profiles, 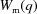, in arbitrary units, *versus* magnitude of the scattering vector, corresponding to the instrumental configurations of Table 1 ▸. The solid lines are least-squares fits, as described in the text, referred to as a polynomial representation of the experimental beam profiles.

Fig. 3 ▸ shows the measured beam intensities of the four instrumental configurations given in Table 1 ▸. A reasonable representation of the experimental beam profiles is in most cases given by the simple trapezoidal function. However, the resolution estimate RES in Table 1 ▸, corresponding to the full trapezoidal beam width, underestimates the actual full beam width somewhat. A more serious effect is that the beam profile corresponding to the highest-resolution configuration in Fig. 3 ▸ is not well captured by the calculations. This is easily remedied because with the measured beam profiles in hand an improved representation can be generated by fitting a suitable function to the data. In this case one could opt for a one-parameter fit to a Gaussian beam profile. However, if we are going to fit the beam profile data, we might as well seek an even better representation. Fig. 4 ▸ shows nonlinear least-squares fits of 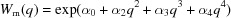 with 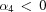. We will refer to this as a polynomial representation, in which 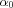
–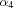 are coefficients determined by the fit. With an algorithm that can handle more complex beam profiles than Gaussian ones, the use of either the trapezoidal or this representation of the beam profile presents no obstacle to determining the smeared model intensity. Smearing calculations were made with these representations of the beam profiles, discretized using 20 wavectors and δ = 0.0001 Å^−1^, yielding 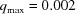
 Å^−1^ (configuration 4), or 30 wavevectors and 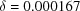
 Å^−1^, leading to 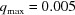
 Å^−1^ (configurations 3 and 26).

**Figure 5:**
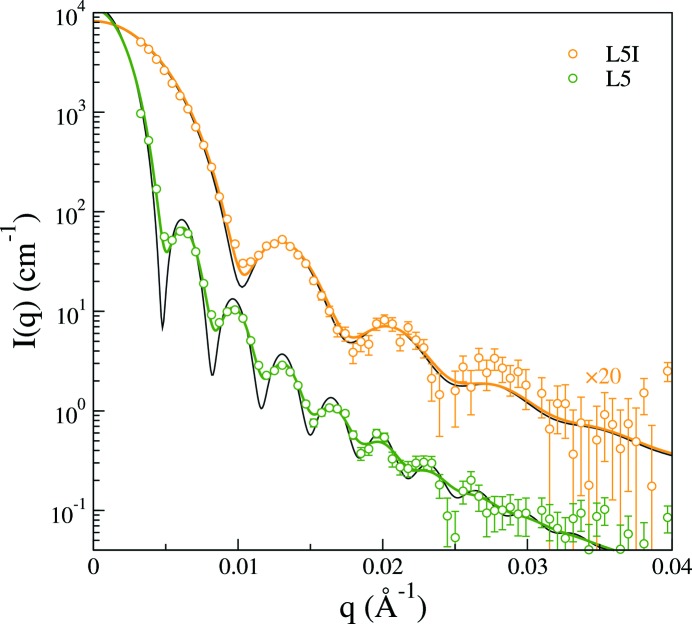
Scattering intensity on absolute scale for samples L5 and L5I as a function of the magnitude of the scattering vector recorded using the SAXS instrument in configuration 4. The (colored) solid lines are model form factors, obtained by least-squares minimization, with smearing included using direct integration of the trapezoidal representation of the beam profile in Fig. 3 ▸. The black solid lines are the corresponding ideal intensities with no instrumental resolution effects included. The results have been shifted vertically for clarity as labeled.

Configuration 4 in Table 1 ▸ yields the best resolution with the smallest beam size on the detector. In Fig. 5 ▸ it is used to study dilute dispersions of the L5 and L5I systems of fluorinated polymer spheres. Form factors for homogeneous spheres with some polydispersity (Aragon & Pecora, 1976 ▸) capture the data relatively well provided smearing effects are taken into account. As shown by the ideal unsmeared form factors included in Fig. 5 ▸, there is severe disagreement between theory and experiment, particularly close to the first minimum of the form factor, if one takes no account of smearing effects. However, agreement between theory and experiment is vastly improved by accounting for smearing using the trapezoidal representation of the primary beam profiles in Fig. 3 ▸. Least-squares minimization between experiment and model leads to a mean radius of 43.4 nm and a polydispersity of 7.7% for the L5I system and a mean radius of 93.2 nm and a polydispersity of 4.9% for the L5 system. This illustrates that making a snap judgment as to the relative polydispersity of the two systems based on the depth and distinctiveness of the first form factor minimum can lead to the wrong conclusion in the presence of instrumental smearing. The figure shows how the instrumental resolution effects cause strong smearing in areas where the intensity changes strongly with *q*, leading to a marked smearing of the first form factor minimum of the L5 system with progressively less smearing of the ensuing minima. Accordingly, it is rather the number of resolved form factor oscillations that is a telltale sign of a low polydispersity (Wilcoxon *et al.*, 1996 ▸).

**Figure 6:**
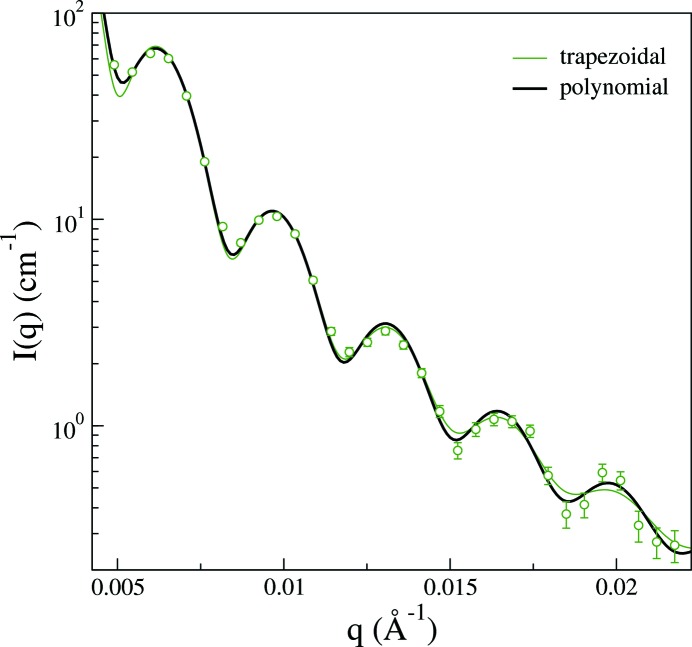
Scattering intensity on absolute scale for sample L5 as a function of the magnitude of the scattering vector recorded using the SAXS instrument in configuration 4. The (colored) solid line is the model form factor, obtained by least-squares minimization, with smearing included using direct integration of the trapezoidal representation of the beam profile in Fig. 3 ▸. The black solid line has been obtained with smearing included using direct integration of the fitted ‘polynomial’ representation of the beam profile in Fig. 4 ▸.

Having performed measurements of the beam profiles there is of course no need to estimate them from the geometry of the experimental setup. Instead, a straightforward fitting of a suitable functional form yields an improved representation, able to capture the slightly wider beams measured in Fig. 4 ▸ compared to the trapezoidal estimates in Fig. 3 ▸. The improvement over the trapezoidal representation is particularly significant for configuration 4. Fig. 6 ▸ shows an enhancement about the scattering intensity from the L5 system using configuration 4, where, in addition to the smeared intensity resulting from the trapezoidal representation of the beam profile, the smeared intensity determined from the polynomial representation of the beam profile is shown. Both models have been optimized through least-squares minimization with respect to the logarithm of the intensity, which was employed in order to get the procedure to better respect the oscillations at high *q*. A conventional least-squares minimization with respect to the intensity leads to a somewhat greater polydispersity and less pronounced oscillations of the model at high *q*. The differences are rather small in Fig. 6 ▸ but systematic in that the polynomial representation of the beam yields a stronger smearing of the first form factor minimum while causing less smearing at larger wavevectors compared to use of the trapezoidal beam profile. This results primarily from the mismatch of the FWHM between the experimental beam and the calculations based on the trapezoidal profile. The extracted parameters also change somewhat. The mean radius obtained is 93.8 nm and the polydispersity is 3.8% with the polynomial representation of the beam, compared to 93.2 nm and a polydispersity of 4.9% with the trapezoidal representation of the beam. The result for the radius is hardly significant but the smaller polydispersity is in better agreement with that obtained from cryo-TEM measurements (Ulama *et al.*, 2014 ▸).

**Figure 7:**
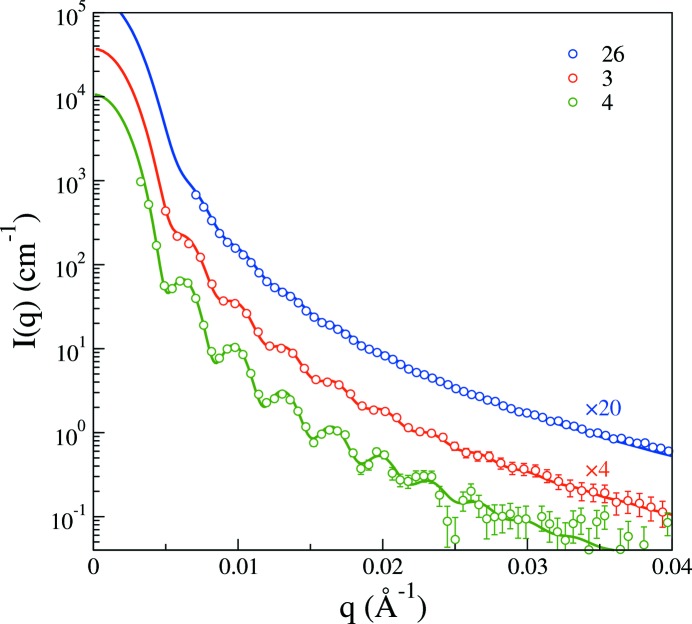
Scattering intensity on absolute scale for the L5 system as a function of the magnitude of the scattering vector and the configuration of the instrument, as labeled. The solid lines are model form factors, determined using least-squares minimization against the scattering data for configuration 4, with smearing included using the ‘polynomial’ representation of the measured beam profiles in Fig. 4 ▸.

For measurements using laboratory-scale SAXS instruments one has to consider the trade-off between resolution and flux. For instance, as configuration 4 is changed to configuration 3 (see Table 1 ▸) more than a factor of 4 in incident intensity is gained. Removing one of the pinholes as done with configuration 26 increases the flux about 50-fold relative to configuration 4. On the other hand, as shown in Fig. 7 ▸ for the latex L5 spheres, the degree of smearing increases quite substantially. Here, we focus on the L5 system, which, with the larger sphere size, exhibits considerable instrumental smearing effects even in the highest-resolution configuration in Fig. 5 ▸. Going to configuration 3, we note that, although the smearing has increased in Fig. 7 ▸, some of the form factor minima remain in the form of clearly observable oscillations. These grow weaker but still remain to some degree when the same sample is measured with the instrument in configuration 26. Using configuration 2 the scattering data are completely smeared, and they have been omitted for this reason. We now lock the parameters at the values obtained from regression of the model with the measurement using configuration 4. In other words, the model curves shown in Fig. 7 ▸ have been obtained from the same form factor only smeared using the polynomial representation of the beam profiles in Fig. 4 ▸ without adjustment of any parameters. As seen, the scattering data can be consistently modeled using this procedure, which gives some credence to modeling efforts involving SAXS data that are rather strongly distorted by instrumental smearing provided a careful analysis of the beam profile and/or resolution function has been made.

## 5. Conclusions   

A new algorithm has been presented in this work that accommodates isotropic but otherwise arbitrary beam profiles in accounting for instrumental resolution effects. It requires only a single cubic spline fit of the theoretical model intensity and reduces computations essentially to determining numerically a single one-dimensional wavevector integral over the beam width. The method has been tested using measurements of latex samples on a laboratory-scale SAXS instrument with pinhole collimation. Beam profiles have been measured and analyzed rather than relying only on theoretical estimates. While simple trapezoidal beam profiles capture the measured profiles reasonably well, the analysis shows that it is worthwhile to obtain better representations of the beams by regression of beam data to some suitable functional form. With measured and analyzed beam profiles in hand, smearing of model intensities is shown to allow for modeling experimental data in a consistent way even under conditions of appreciable instrumental smearing.

## Figures and Tables

**Table 1:** Instrumental configurations in terms of the diameters of the first, second and third apertures (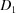
–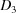), the distances between the first and second apertures (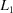), second and third apertures (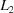) and third aperture and sample (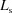), and the sample-to-detector distance (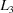), all in units of millimetres, and resolution (RES) in units of Å^−1^ For configuration 26 the second aperture is absent and 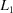 refers to the distance between the first and third aperture.

Configuration	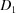	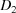	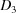	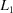	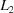	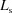	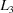	RES
2	0.4	0.3	0.54	750	480	150	441.7	0.0046
3	0.3	0.15	0.34	750	480	150	1041.7	0.0019
4	0.2	0.10	0.24	750	480	150	1491.7	0.0011
26	0.6	–	0.46	1230	–	150	1491.7	0.0026

## Appendix A Effects of detector resolution

In §3.1 the resolution function of the detector was not shown explicitly. The reason for this is that it is compounded with the effect of finite collimation in the measured beam profile  in equation (1) (Pedersen & Riekel, 1991 ▸; Pauw, 2013 ▸). To see how this comes about, we write

where  and  contain the separate effects of finite collimation and detector resolution, respectively (Ramakrishnan, 1985 ▸). Fourier transforming both sides of equation (16) yields . We can make equations (1) and (16) consistent with one another by identifying

which transforms as

Changing the order of integration leads to

where  is the radially averaged detector resolution function. In terms of resolution functions the above is consistent with the definition of a total resolution function as , with , a one-dimensional analogue of a result given by Pedersen & Riekel (1991 ▸). It follows that  is the measured beam profile, which is given as a convolution of the finite but otherwise clean (as in being unaffected by detector resolution) beam profile  and the detector resolution .
